# Accuracy of the endoscopic evaluation of esophageal involvement in esophagogastric junction cancer

**DOI:** 10.1016/j.amsu.2021.102590

**Published:** 2021-07-30

**Authors:** Takeshi Sakai, Hiroshi Ichikawa, Takaaki Hanyu, Kenji Usui, Yosuke Kano, Yusuke Muneoka, Takashi Ishikawa, Yoshifumi Shimada, Jun Sakata, Toshifumi Wakai

**Affiliations:** Division of Digestive and General Surgery, Niigata University Graduate School of Medical and Dental Sciences, 1-757 Asahimachi-dori, Chuo-ku, Niigata City, Niigata, 951-8510, Japan

**Keywords:** Esophageal involvement length, Esophagogastric junction, Adenocarcinoma, Mediastinal lymph node metastasis, Lymph node dissection, EIL, esophageal involvement length, MLNs, mediastinal lymph nodes, EGJ, esophagogastric junction, OR, odds ratio, CI, confidence of interval

## Abstract

**Background:**

Esophageal involvement length (EIL) is a promising indicator of metastasis or recurrence in mediastinal lymph nodes (MLNs) in adenocarcinoma of the esophagogastric junction (EGJ). This study aimed to elucidate the accuracy of the preoperative endoscopic evaluations of EIL and its clinical significance in this disease.

**Materials and methods:**

In total, 75 patients with Siewert type II (N = 53) or III (N = 22) adenocarcinoma of the EGJ, who underwent surgical resection without preoperative therapy between 1995 and 2016 were enrolled. We retrospectively examined the accuracy of the preoperative endoscopic evaluations of EIL (preoperative EIL), compared to the pathologically evaluated EIL. Finally, we investigated the association between preoperative EIL and metastasis or recurrence in MLNs.

**Results:**

The accuracy of the preoperative EIL within a 1-cm interval was only 53.3%. Among patients with discordance between the pre- and postoperative evaluations, 68.6 % had the underestimation in the preoperative EIL. pN1–3 (OR = 5.85, 95% CI: 1.03–33.17) and undifferentiated histologic type (OR = 2.52, 95% CI: 0.89–7.14) were potential risk factors for the discordance. Regarding metastasis or recurrence in MLNs, preoperative EIL of 2–3 cm (OR = 10.41, 95% CI: 1.35–80.11) and >3 cm (OR = 8.33, 95% CI: 1.09–63.96) were independent predictors.

**Conclusion:**

Although the accuracy of the endoscopic evaluations of EIL is insufficient with many underestimations, EIL should be assessed in preoperative staging because of significant predictive power for metastasis or recurrence in MLNs.

## 1 Introduction

The incidence of adenocarcinoma of the esophagogastric junction (EGJ) has risen, not only in Western countries but also in eastern Asia [1,2]. Similar to esophageal and gastric cancers, surgical resection with lymph node dissection is the mainstay of curative treatment for adenocarcinoma of the EGJ. As this tumor has esophageal involvement and the risk of metastasis in mediastinal lymph nodes (MLNs), ensuring a negative proximal margin and determining the extent of lymph node dissection are essential to perform curative surgery successfully. The Siewert classification, which is the most commonly used classification system, differentiated the three distinct entities of adenocarcinoma within a 5 cm proximal and distal distance to the EGJ based on the location of epicenter as follows; more than 1 cm proximal to the EGJ (type I), within 1 cm proximal and 2 cm distal to the EGJ (type II), and more than 2 cm distal to the EGJ (type III) [3]. Siewert type I tumor is usually resected by transthoracic subtotal esophagectomy with a mediastinal lymph node dissection for esophageal cancer. However, the extent of esophageal resection and mediastinal lymph node dissection for Siewert type II or III tumors are not standardized; these are determined on a case by case basis.

Previous studies suggested that lymphatic drainage from the gastric cardia to mediastinal paraesophageal lymph nodes was rare [4]. However, an esophageal wall had longitudinal lymphatic vessels which were connected to the mediastinal lymph nodes, especially the right upper paratracheal nodes [5,6]. Thus, esophageal involvement in EGJ adenocarcinoma was considered to be correlated with the incidence of metastasis in MLNs. Recent clinical studies revealed that esophageal involvement length (EIL) in Siewert type II tumor was a reliable indicator of metastasis or recurrence in MLNs [[7], [8], [9]]. Therefore, EIL evaluation is vital to determine optimal surgical procedures.

To acquire biopsy specimens for pathological confirmation of a malignant tumor, gastrointestinal endoscopy is routine in the clinical examination of a suspected adenocarcinoma of the EGJ. EIL is also mainly evaluated by endoscopy, but its accuracy has not been fully investigated. We examined the accuracy of the preoperative endoscopic evaluations of EIL, and investigated the significance of EIL in metastasis or recurrence in MLNs.

## 2 Materials and methods

### 2.1 Patients

Ninety-one patients with Siewert type II or III adenocarcinoma underwent surgical resection at our institution between 1995 and 2016. Of these, we enrolled 75 patients in this single-institutional retrospective study, excluding seven who underwent endoscopic resection before surgery and nine who received preoperative chemotherapy. This study has been reported in line with the Strengthening the Reporting of Cohort Studies in Surgery (STROCSS) criteria [10]. This study was registered at Research Registry (researchregistry6859, https://www.researchregistry.com/browse-the-registry#home/registrationdetails/60aE0533695557001ef111f3/). The clinicopathological characteristics are described in Table 1. This study was conducted in accordance with the provisions of the Declaration of Helsinki, and the Ethics Committee at the Niigata University approved the study protocol (#2018–0137). The need for written informed consent was waived, and brief information on this study was disclosed on the Niigata University website to guarantee a patients’ opportunity to refuse their participation in the study (opt-out method).

**Table 1:** Clinicopathologic characteristics (N = 75).

**Variable**	**No. of patients (%) or median (range)**
Age (years)	69 (33–86)
Gender	
Male	62 (82.7)
Female	13 (17.3)
Surgical approach	
Transthoracic and abdominal	1 (1.3)
Abdominal transhiatal	39 (52.0)
Abdominal	35 (46.7)
Extent of esophagectomy	
Subtotal	11 (14.7)
Lower	64 (85.3)
Extent of gastrectomy	
Total	62 (82.7)
Proximal	13 (17.3)
Extent of lymph node dissection	
Upper, middle, lower mediastinal and abdominal	1 (1.3)
Lower mediastinal and abdominal	36 (48.0)
Abdominal	38 (50.7)
Siewert type (preoperative)	
II	52 (69.3)
III	23 (30.7)
Tumor length (preoperative)	
≤5.0 cm	28 (37.3)
>5.0 cm	47 (62.7)
Histologic type (preoperative biopsy)	
Differentiated	48 (64.0)
Undifferentiated	27 (36.0)
Clinical T stage	
cT1	14 (18.7)
cT2	7 (9.3)
cT3	52 (69.3)
cT4	2 (2.7)
Clinical N stage	
cN0	41 (54.7)
cN1	24 (32.0)
cN2	7 (9.3)
cN3	3 (4.0)
Clinical M stage	
cM0	69 (92.0)
cM1	6 (8.0)
Siewert type (postoperative)	
II	53 (70.7)
III	22 (29.3)
Tumor length (postoperative)	
≤5.0 cm	27 (36.0)
>5.0 cm	48 (64.0)
Histologic type (postoperative)	
Differentiated	44 (58.7)
Undifferentiated	31 (41.3)
Pathologic T stage	
pT1	17 (22.7)
pT2	4 (5.3)
pT3	49 (65.3)
pT4	5 (6.7)
Pathologic N stage	
pN0	24 (32.0)
pN1	20 (26.7)
pN2	17 (22.7)
pN3	14 (18.7)
Pathologic M stage	
pM0	56 (74.7)
pM1	19 (25.3)
Lymphatic invasion	
Absence	23 (30.7)
Presence	52 (69.3)
Venous invasion	
Absence	46 (61.3)
Presence	29 (38.7)
Proximal margin	
Negative	71 (94.7)
Positive	4 (5.3)

### 2.2 Evaluation of EIL

We determined preoperative EIL by the endoscopic evaluation before surgery. EGJ was defined using the lowest mark of the palisade blood vessels of the esophagus and the proximal margin of the gastric mucosal folds as markers per the Prague Criteria for the grading of Barrett's esophagus and the Minimal Standard Terminology for Gastrointestinal Endoscopy ver. 3.0 [11,12]. EIL was determined as the distance from the EGJ to the proximal edge of the tumor. Three gastrointestinal surgeons specialized in the upper gastrointestinal surgery and endoscopy independently reviewed the multiple endoscopic images showing the tumor and EGJ in color for each patient. The EIL was estimated by using criterion as follow: 1-cm interval (≤1 cm, 1–2 cm, 2–3 cm, or >3 cm) according to their experience of endoscopy. In the case of the discordance, final results were established according to the reviewers' reassessment and discussion. Preoperative Siewert type was also determined by ascertaining the location of the tumor epicenter via endoscopic findings. Postoperative EIL was pathologically evaluated in the surgical specimen by measuring the distance from EGJ to the proximal edge of the tumor infiltration, including the lymphovascular invasion.

### 2.3 Cancer staging and surgery

The cancer stage was determined based on the 7th edition of the International Union Against Cancer TNM Classification of Malignant Tumors [13]. The lymph node station numbers were defined by the Japanese Classification of Esophageal Cancer (11th edition) [14]. The MLNs were classified into three groups as follows: lower MLNs included stations 110, 111, and 112, middle MLNs included stations 107, 108, 109L and 109R, and upper MLNs included stations 105, 106recL and 106recR. Surgical procedures were selected based on the surgeon's decision, considering the tumor location, esophageal involvement, and the preoperative conditions of patients. Details about the surgical approach and the extent of esophagectomy and gastrectomy are shown in Table 1. Upper, middle, lower mediastinal, and abdominal lymph node dissections were performed in 1 (1.3%) patient, and lower mediastinal and abdominal lymph node dissections were performed in 36 (48.0%) patients. All patients were followed up at 3-months intervals after esophagectomy, with routine physical and laboratory examinations conducted. Computed tomography was performed every six months to detect tumor recurrence.

### 2.4 Outcomes and statistical analysis

The primary outcome was the accuracy of endoscopic evaluations of EIL, and the secondary outcomes were risk factors for the discordance between the pre- and postoperative evaluations of EIL and for metastasis or recurrence in MLNs. The differences between the two groups were assessed using Fisher's exact test for categorical variables in the univariate analysis. Multivariate analysis using a logistic regression model was performed to identify the independent risk factors, calculating the odds ratio (OR) and 95% confidence interval (CI). Factors with *P* < 0.05 in the univariate analysis were included in the multivariate analysis. All statistical analyses were performed using the PASW Statistics 24 software package (SPSS Japan Inc, Tokyo, Japan). *P*-values <0.05 (two-tailed) were considered statistically significant.

## 3 Results

### 3.1 Accuracy of the endoscopic evaluations of EIL

Details of the pre- and postoperative evaluations of EIL are shown in Table 2. According to the endoscopic evaluations, preoperative EIL ≤1 cm and >3 cm were identified in 43 (57.3%) and 6 (8.0%) patients, respectively. In contrast, the number of patients with EIL ≤1.0 cm was 36 (48.0%), and those with EIL >3.0 cm was 15 (20.0%) in the postoperative evaluations. Among 75 patients, four (5.3%) had no definitive postoperative EIL data in the surgical specimen due to tumor positive proximal margin. However, the length of the lower esophagus removed was more than 3.0 cm in three of four patients. Thus, postoperative EIL was determined to be >3.0 cm in these patients. In the remaining one (1.3%) patient with 1–2 cm preoperative EIL, postoperative EIL was estimated to be at least >2.0 cm because the length of the lower esophagus removed was 2.8 cm. Among 35 (46.6%) patients with discordance between the pre- and postoperative evaluations of EIL (discordant group), 24 (68.6%) had the underestimation, and only 11 (31.4%) had the overestimation in the preoperative endoscopic evaluation of EIL. There was concordance between the pre- and postoperative EIL evaluations within a 1-cm interval in the remaining 40 patients (concordant group), but the accuracy was only 53.3%.

**Table 2:** Esophageal involvement length in the endoscopic evaluations and surgical specimen.

		**Postoperative EIL in the surgical specimen**					
		≤**1.0 cm**	**1.1**–**2.0 cm**	**2.1**–**3.0 cm**	>**3.0 cm**	**NA**	**Total (%)**
**Preoperative EIL in the endoscopic evaluations**	≤**1 cm**	27	9	2	5	0	43 (57.3)
	**1**–**2 cm**	9	7	2	1	1*	20 (26.7)
	**2**–**3 cm**	0	1	1	4	0	6 (8.0)
	>**3 cm**	0	0	1	5	0	6 (8.0)
	**Total (%)**	36 (48.0)	17 (22.7)	6 (8.0)	15 (20.0)	1 (1.3)	75 (100)

EIL, esophageal involvement length; NA, not available.

* One patient with 1–2 cm of preoperative EIL had no definitive data of postoperative EIL in the surgical specimen due to tumor positive proximal margin. Postoperative EIL was estimated to be at least >2.0 cm because the length of the lower esophagus removed was 2.8 cm.

### 3.2 Risk factors for the discordance of the evaluations of EIL

**Fig. 1:**
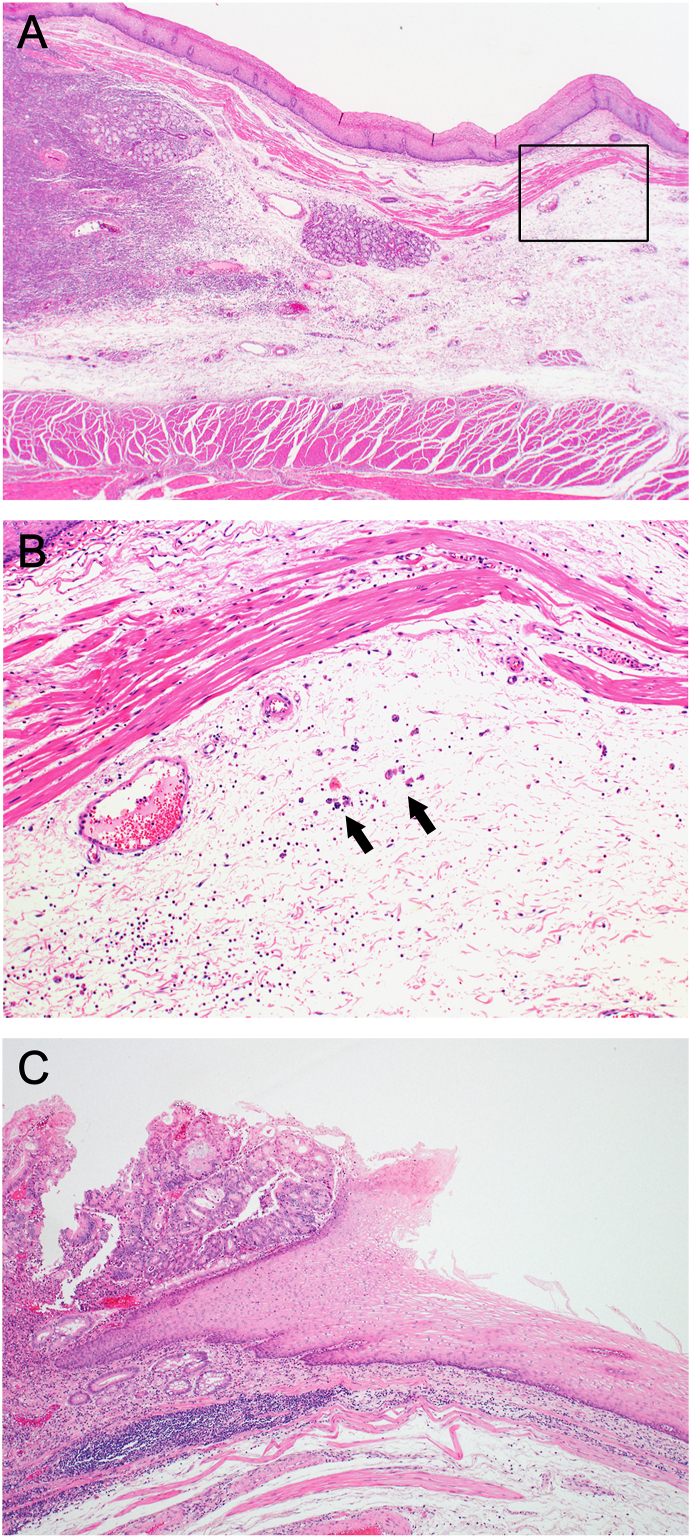
Representative histological images of esophageal involvement. (A) The undifferentiated type tumor with submucosal infiltration in the proximal esophageal wall in patients with under-estimation of esophageal involvement length (EIL) in preoperative endoscopic evaluation. The boxed region is magnified in Figure B (H.E., original magnification × 40). (B) Poorly cohesive cells (arrows) are infiltrating into the submucosa of the proximal esophageal wall (H.E., original magnification × 200). (C) The differentiated type tumor with the concordant evaluation of EIL has a clear margin without submucosal infiltration in the proximal esophageal wall (Fig. 1C).

We compared clinicopathological features between the concordant (N = 40) and discordant groups (N = 35). The undifferentiated histologic type tumor was more frequently observed in the discordant group than in the concordant group (57.1% vs. 27.5%, *P* = 0.01). There were also significant differences between the two groups in the frequency of pT2–4 (91.4% vs. 65.0%, *P* = 0.01) and pN1–3 (88.6% vs. 50.0%, *P* < 0.01). In the multivariate analysis, pN1–3 (OR = 5.85, 95% CI: 1.03–33.17, *P* = 0.046) and undifferentiated histologic type (OR = 2.52, 95% CI: 0.89–7.14, *P* = 0.08) were potential independent risk factors for the observed discordance in the evaluations of EIL (Table 3). Fig. 1A and B shows the representative histological images of the undifferentiated type tumor with submucosal infiltration of the proximal esophageal wall in patients with underestimation of EIL in the preoperative endoscopic evaluations. In contrast, the differentiated type tumors, in which concordance was observed in the evaluations of EIL, had clear margins without submucosal infiltration of the proximal esophageal wall (Fig. 1C).

**Table 3:** Risk factors for the discordance of EIL between the endoscopic evaluations and surgical specimen.

**Variable**		**No. of patients (%)**		**Univariate**	**Multivariate**		
		**Concordant (N** = **40)**	**Discordant (N** = **35)**	***P***	**OR**	**95 % CI**	***P***
Age (years)							
<70		24 (60.0)	15 (42.9)	0.168			
≥70		16 (40.0)	20 (57.1)				
Gender							
Male		31 (77.5)	31 (88.6)	0.238			
Female		9 (22.5)	4 (11.4)				
Siewert type (postoperative)							
II		26 (65.0)	27 (77.1)	0.313			
III		14 (35.0)	8 (22.9)				
Tumor length (postoperative)							
≤5.0 cm		17 (42.5)	10 (28.6)	0.237			
>5.0 cm		23 (57.5)	25 (71.4)				
Histologic type (postoperative)							
Differentiated		29 (72.5)	15 (42.9)	0.011	1.00		
Undifferentiated		11 (27.5)	20 (57.1)		2.52	0.89–7.14	0.082
Pathological T stage							
pT1		14 (35.0)	3 (8.6)	0.011	1.00		
pT2–4		26 (65.0)	32 (91.4)		1.12	0.16–8.13	0.908
Pathological N stage							
pN0		20 (50.0)	4 (11.4)	<0.001	1.00		
pN1–3		20 (50.0)	31 (88.6)		5.85	1.03–33.17	0.046
Pathological M stage							
cM0		32 (80.0)	24 (68.6)	0.296			
cM1		8 (20.0)	11 (31.4)				
Lymphatic invasion							
Absence		13 (32.5)	10 (28.6)	0.804			
Presence		27 (67.5)	25 (71.4)				
Venous invasion							
Absence		26 (65.0)	20 (57.1)	0.635			
Presence		14 (35.0)	15 (42.9)				

EIL, esophageal involvement length; OR, odds ratio; CI, confidence interval.

### 3.3 Predictors for metastasis or recurrence in MLNs

The median follow-up time after surgery was 98 months (range, 53–183) in surviving patients. Among 75 patients, 11 had the recurrence or metastasis in MLNs. We compared the preoperatively evaluated clinicopathological features between patients without metastasis or recurrence in MLNs (absent group, N = 64) and those with it (present group, N = 11) (Table 4). The proportion of patients with longer EIL in the preoperative endoscopic evaluations was significantly higher in the present group than in the absent group (*P* = 0.004). Tumor length >5.0 cm was more frequently observed in the present group than in the absent group (90.9% vs. 57.8%, *P* = 0.045). Multivariate analysis, including preoperative EIL and tumor length as co-factors, demonstrated that preoperative EIL was an independent predictor for metastasis or recurrence in MLNs with the high risk in patients with EIL of 2–3 cm (OR = 10.41, 95% CI: 1.35–80.11, *P* = 0.024) and EIL >3 cm (OR = 8.33, 95% CI: 1.09–63.96, *P* = 0.041).

**Table 4:** Predictors for metastasis or recurrence in the mediastinal lymph nodes.

**Variable**	**No. of patients (%)**		**Univariate**	**Multivariate**		
	**Met or Rec in MLNs**		***P***	**OR**	**95 % CI**	***P***
	**Absence (N** = **64)**	**Presence (N** = **11)**				
Age (years)						
<70	31 (48.4)	8 (72.7)	0.195			
≥70	33 (51.6)	3 (27.3)				
Gender						
Male	52 (81.3)	10 (90.9)	0.677			
Female	12 (18.7)	1 (9.1)				
Siewert type (preoperative)						
II	43 (67.2)	9 (81.8)	0.486			
III	21 (32.8)	2 (18.2)				
Tumor length (preoperative)						
≤5.0 cm	27 (42.2)	1 (9.1)	0.045	1.00		
>5.0 cm	37 (57.8)	10 (90.9)		4.36	0.47–40.41	0.194
Histologic type (preoperative)						
Differentiated	43 (67.2)	5 (45.5)	0.188			
Undifferentiated	21 (32.8)	6 (54.5)				
Clinical T stage						
cT1	14 (21.9)	0 (0)	0.112			
cT2–4	50 (78.1)	11 (100)				
Clinical N stage						
cN0	37 (57.8)	4 (36.4)	0.209			
cN1–3	27 (42.2)	7 (63.6)				
Clinical M stage						
cM0	59 (92.2)	10 (90.9)	1.000			
cM1	5 (7.8)	1 (9.1)				
EIL (preoperative)∗						
≤1 cm	40 (62.5)	3 (27.3)	0.004	1.00		
1–2 cm	18 (28.1)	2 (18.1)		1.30	0.19–8.73	0.785
2–3 cm	3 (4.7)	3 (27.3)		10.41	1.35–80.11	0.024
>3 cm	3 (4.7)	3 (27.3)		8.33	1.09–63.96	0.041

EIL, esophageal involvement length; Met, metastasis; Rec, recurrence; MLNs, mediastinal lymph nodes; OR, odds ratio; CI, confidence interval.

∗ EIL was evaluated by the preoperative endoscopy.

### 3.4 Distribution of metastasis or recurrence in MLNs

Finally, we reviewed the distribution of metastasis or recurrence in MLNs according to the preoperative endoscopic evaluations of EIL (Table 5). The overall metastasis or recurrence rate was 14.7%, with 12.0% in the lower, 1.3% in the middle, and 1.3% in the upper MLNs. The metastasis or recurrence rate in the lower MLNs was 33.0% in patients with EIL of 2–3 cm and with EIL >3 cm. The metastasis or recurrence in the middle and upper MLNs was observed in one patient with EIL of 2–3 cm (16.7%) and in one patient with EIL >3 cm (16.7%), respectively.

**Table 5:** Metastasis or recurrence rates in the mediastinal lymph nodes according to preoperative EIL in the endoscopic evaluations.

**Preoperative EIL**	**Metastasis or recurrence rates in the MLNs**			
	Total, % (n/N)*	Lower, % (n/N)*	Middle, % (n/N)*	Upper, % (n/N)*
≤1 cm	7.0 (3/43)	7.0 (3/43)	0.0 (0/43)	0.0 (0/43)
1–2 cm	10.0 (2/20)	10.0 (2/20)	0.0 (0/20)	0.0 (0/20)
2–3 cm	50.0 (3/6)	33.3 (2/6)	16.7 (1/6)	0.0 (0/6)
>3 cm	50.0 (3/6)	33.3 (2/6)	0.0 (0/6)	16.7 (1/6)
Total	14.7 (11/75)	12.0 (9/75)	1.3 (1/75)	1.3 (1/75)

EIL, esophageal involvement length; MLNs, mediastinal lymph nodes.

* “n” and “N” indicate the number of patients with the metastasis or recurrence and that of patients who classified into each EIL category, respectively.

## 4 Discussion

In this study, we found that the preoperative evaluations of EIL, using the criterion of within a 1-cm interval, had low reliability, with only a 53.3% accuracy. A previous study also reported a low accuracy of endoscopic evaluations of EILs within a 2-cm interval, with 74.1% [15]. According to the recent nationwide prospective study, which investigated the distribution of lymph node metastasis in Siewert type II EGJ cancer, the diagnostic accuracies of the endoscopic evaluations concerning whether EIL was more than 2.0 cm and more than 4.0 cm were shown as 79.8% and 95.6%, respectively [9]. Therefore, the rough evaluation of EIL with endoscopy could be feasible. Of the patients in which discordance between pre- and postoperative evaluations of EIL were observed, the majority were underestimations of EIL in this study. Surgeons have to take caution to determine the lengths of esophageal resection, considering the underestimations of EIL by preoperative endoscopy.

We identified histologically undifferentiated type as one of the potential risk factors for the discordance between pre- and postoperative evaluations of EIL. As shown in Fig. 1, the histologically undifferentiated tumor had tumor infiltration within the submucosal layer, which resulted in the underestimation of EIL by endoscopy. The pathological presence of lymph node metastasis (pN1−3) was also shown as a risk factor for the observed discordance. Histological confirmation with the frozen-section analysis of the proximal margin has to be carried out in the clinical node positive undifferentiated tumor. Furthermore, we should prepare for the transthoracic approach considering additional resection of the thoracic esophagus with mediastinal lymph node dissection, and safe reconstruction in patients with these risk factors.

EIL is gathering attention as a potential indicator of metastasis or recurrence in MLNs [7,8]. We demonstrated that patients with EIL of 2–3 cm and EIL >3 cm were the high-risk population of metastasis or recurrence in MLNs. Although the present study was a retrospective one with a small number of subjects, EIL was confirmed to be the essential factor for preoperative assessment to decide the indication of mediastinal lymph node dissection. In this study, the rate of metastasis or recurrence in lower MLNs was 33.3% in patients with EIL of 2–3 cm and with EIL >3 cm, which was remarkably higher than other patient groups. Therefore, at least the lower MLNs should be dissected in patients with EIL >2 cm. Unfortunately, we could not establish the definitive strategy for the upper and middle mediastinal lymph node dissection according to our limited data. A recent large scale prospective study revealed that the rate of metastasis in station 106recR (right recurrent laryngeal nerve nodes) exceeded 10% and that in the middle MLNs was around 7% in patients with EIL >4.0 cm [9]. Thus, subtotal esophagectomy to dissect the upper and middle mediastinal lymph nodes is recommended for patients with EIL >4.0 cm.

Gastrointestinal surgeons have to select the optimal surgical approach for adenocarcinoma of the EGJ, considering the length of esophageal resection to ensure a negative proximal margin, the extent of lymph node dissection, and safe gastrointestinal reconstruction. According to the recent impressive research, EIL is highlighted as a promising factor in determining not only the length of esophageal resection but also the extent of mediastinal lymph node dissection. Thus, accurate preoperative evaluation of EIL is vital in determining the optimal surgical treatment strategy in this disease. However, there is a paucity of data about the accuracy of the preoperative endoscopic evaluations of EIL, which is a routine clinical examination for gastrointestinal cancers. This study focused on the accuracy of the preoperative evaluations of EIL. Besides, we confirmed the association between the preoperative evaluations of EIL and metastasis or recurrence in MLNs.

The present study has the following limitations. This study had a small number of enrolled patients because of single-institutional design. However, 75 of 91 consecutive patients with Siewert type II or III adenocarcinoma who underwent esophagectomy without preoperative chemotherapy were enrolled. This high inclusion rate (82.4%) contributes to reducing the potential selection bias. The evaluation of metastasis in MLNs was insufficient because the dissection of MLNs was not performed in 52.0% (39/75) of enrolled patients. We ensured the possibility of metastasis in MLNs by including postoperative recurrence in MLNs with a sufficient follow-up period into the analysis. Therefore, we believed that our findings are clinically informative for preoperative diagnosis and decision making for the surgical approach in Siewert type II and III adenocarcinoma.

## 5 Conclusions

The accuracy of the preoperative endoscopic evaluations of EIL in adenocarcinoma of the EGJ is insufficient and is accompanied by many underestimations. The presence of lymph node metastases and histologically undifferentiated type are potential risk factors for the discordance between the pre- and postoperative evaluations of EIL. Despite the low accuracy of the endoscopic evaluations, EIL should be assessed in the preoperative staging because of significant predictive power for metastasis or recurrence in MLNs.

## Sources of funding

None.

## Ethical approval

The Ethics Committee at the Niigata University approved the study protocol (#2018–0137).

## Consent

Not applicable.

## Author contribution

Study design: Takeshi Sakai, Hiroshi Ichikawa. Data collection: Takeshi Sakai, Hiroshi Ichikawa and Yosuke Kano.Reviewing gastrointestinal endoscopic findings: Hiroshi Ichikawa, Takaaki Hanyu and Kenji Usui. Data analysis: Takeshi Sakai, Hiroshi Ichikawa. Writing the paper: Takeshi Sakai, Hiroshi Ichikawa. Supervised the study design and data analysis: Yusuke Muneoka, Takashi Ishikawa. Supervised the whole study: Yoshifumi Shimada, Jun Sakata and Toshifumi Wakai. Final approval of the version to be submitted: All authors.

## Registration of research studies

1. Name of the registry: Accuracy of the endoscopic evaluation of esophageal involvement in esophagogastric junction cancer.

2. Unique Identifying number or registration ID: researchregistry6859.

3. Hyperlink to your specific registration (must be publicly accessible and will be checked): https://www.researchregistry.com/browse-the-registry#home/registrationdetails/60ae0533695557001ef111f3/

## Guarantor

Hiroshi Ichikawa is the guarantor of this study.

## Provenance and peer review

Not commissioned, externally peer-reviewed.

## Declaration of competing interest

The authors have no conflicts of interest.
